# Spinal arthritis in invasive cane toads is linked to rate of dispersal as well as to latitude

**DOI:** 10.1038/s41598-019-50314-w

**Published:** 2019-09-27

**Authors:** Gregory P. Brown, Lin Schwarzkopf, Ross A. Alford, Deborah Bower, Richard Shine

**Affiliations:** 1 Department of Biological Sciences, Macquarie University, New South Wales, 2109 Australia; 2 College of Science and Engineering, James Cook University, Townsville, Queensland 4811 Australia; 3 School of Environmental and Rural Science, University of New England, Armidale, New South Wales, 2351 Australia

**Keywords:** Conservation biology, Invasive species

## Abstract

Initial research on the spread of cane toads (*Rhinella marina*) through tropical Australia reported a high incidence of spinal arthritis (spondylosis) in toads at the invasion front (where toads disperse rapidly), but not in areas colonized earlier (where toads are more sedentary). The idea that spondylosis was a cost of rapid dispersal was challenged by wider spatial sampling which linked rates of spondylosis to hot (tropical) climates rather than to dispersal rates. Here, the authors of these competing interpretations collaborate to reinterpret the data. Our reanalysis supports both previous hypotheses; rates of spondylosis are higher in populations established by fast-dispersing toads, and are higher in tropical than in temperate environments; they are also higher in larger toads. The functional reason for climatic effects is unclear, but might involve effects on the soil-living bacteria involved in the induction of spondylosis; and/or may reflect higher movement (as opposed to dispersal) or more pronounced dry-season aggregation rates of toads in tropical conditions.

## Introduction

Following their release in northeastern Australia in 1935, cane toads dispersed at increasing speeds to the west (through the tropics)^1–4^. This dramatic acceleration of rates of dispersal was accompanied by changes in many phenotypic traits^5–9^. In 2007, spondylosis (enlarged lesions on the spinal column, associated with the bacterium *Brucella* [*Ochrobactrum*] spp.) appeared to be more common in cane toads at the tropical invasion front than in conspecifics from longer-colonized areas (where the invasion was moving less rapidly at the time of toad arrival)^1,2,10^. The apparently high incidence of spondylosis at the invasion front (and especially in large, long-legged individuals) was attributed to the high dispersal rates of such animals.

That interpretation was challenged by Bower *et al*.^3^, based on an analysis of >2,400 field-collected toads from 25 sites spanning the species’ Australian invasion range. Bower *et al*.^3^ reported a strong positive association between spondylosis and latitude, and a weak negative correlation between the prevalence of spondylosis and year of population establishment; and concluded that overall, spondylosis in cane toads may be driven by local climatic factors (as reflected by latitude) rather than by invasion history. The two sets of authors have now joined forces to reinvestigate the large data set of Bower *et al*.^3^, and specifically to resolve the contrasting conclusions that rates of spondylosis are either correlated with population dispersal rates^1,2^ or latitude^3^.

## Methods and Results

### Statistical analyses

We excluded one outlier^11^: a small sample (20 toads) from Hughenden in western Queensland, midway through the current invasion range, that had a prevalence of spinal arthritis more than double that of any other population, and five times higher than the mean prevalence across all populations. Excluding this sample did not qualitatively modify any of our conclusions. Unlike the original analysis, we also included site (i.e., sampling location) as a random variable. Importantly, we also recoded one variable. Although Bower *et al*.^3^ used “date since population establishment” as a proxy for invasion speed, the hypothesis that spondylosis is induced by rapid dispersal invokes invasion speed rather than “time since toad arrival” as the critical factor. The correlation between arrival times and invasion speeds is non-linear in tropical Australia (because the rate of toad dispersal has accelerated dramatically^4^) and non-existent at the southern invasion front (where toads of all populations disperse slowly^4^). To test the putative link between dispersal rate and spondylosis, we should examine the relationship between the prevalence of spondylosis and rates of dispersal at the time that toads arrived at each site. Such rates decline post-invasion^12^, but the genetics of each population will be strongly influenced by the attributes (including, dispersal-relevant traits) of their founders^13^.

We extracted data on rates of toad dispersal at the time each population was founded from the analyses of this trait by Urban *et al*.^4^. We fit a generalized linear mixed model using the function glmer from package lme4 in R^14^ that included the presence or absence of spondylosis as a binomial response, and snout-urostyle length, latitude, our estimates of speed of movement at time of toad arrival, all two-way interactions between these covariates, and their three-way interaction as independent variables. Each of the independent variables was column-standardized (Z_i_ = [X_i_ − (mean of all X_i_)]/SD of all X_i_) to place them on a common scale. The model also included site as a random effect.

**Figure 1:**
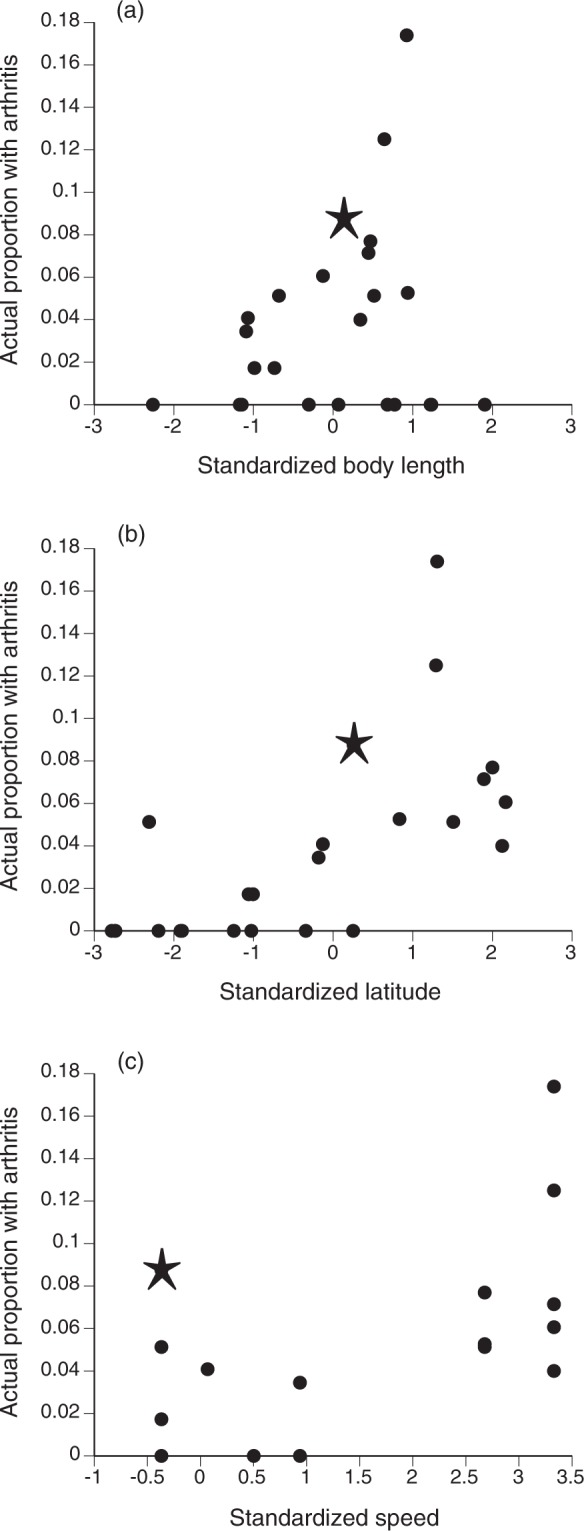
The prevalence of spinal arthritis (spondylosis) in populations of the invasive cane toad (*Rhinella marina*), as a function of (**a**) standardized body size (snout-urostyle length) of toads per population, (**b**) standardized latitude of each sampled site, and (**c**) the standardized speed at which the toad invasion was travelling as it arrived at each site. Each point shows a single site; the asterisk shows Townsville (which had by far the largest sample size). The Figure shows column standardized mean values for each trait for each population, for clarity, but the statistical analyses reported in the text used data for individual toads (with location included as a random effect). Latitudes are standardized, such that positive numbers represent lower (tropical) latitudes. The analysis omits one outlying point (Hughenden, Queensland – 40% of n = 18 toads with spondylosis, invasion speed 25–30 km/yr). Data from Bower *et al*.^3^.

An ANOVA (using the function Anova from package car^15^) indicated that all three of the main effects and the interaction between snout-urostyle length and invasion speed were significant; geographic variation in the prevalence of spondylosis was strongly linked to all three variables that have been suggested as predisposing toads towards this crippling condition. First, spondylosis was more common in larger toads (main effect of snout-urostyle length, χ^2^ = 12.597, 1 df, P = 0.0004; Fig. 1a), as reported by both previous analyses. Spondylosis was also more common at lower latitudes (more tropical sites) than in higher-latitude (temperate-zone) sites (main effect of latitude, χ^2^ = 11.231, 1 df, P = 0.0008; Fig. 1b). Finally, spondylosis was more common in populations that had been founded by faster-dispersing toads (main effect of speed at time of arrival, χ^2^ = 8.279, 1 df, P = 0.0040; Fig. 1c), as reported by Brown *et al*.^1^ and Shilton *et al*.^2^, but opposite to the pattern reported by the Bower *et al*. paper^3^. In addition, after controlling for latitude, the effect of body size on spondylosis was highest among toads from populations founded by faster-dispersing individuals (interaction between snout-urostyle length and speed, χ^2^ = 4.164, 1 df, P = 0.0414). That is, the increased incidence of arthritis in toads from faster-moving populations was most evident among the largest individuals.

## Discussion

The link between spinal arthritis and body size is straightforward; large toads are older and thus have had longer to develop this condition; and also, a larger body places more stress on the spinal column^2,16^. Similarly, we expect higher rates of spondylosis in more active (e.g., rapidly dispersing) individuals (as seen in degenerative forms of arthritis^17^), consistent with the original speculations about the causation of this trait. The causal link between spondylosis and tropical conditions is more ambiguous, however plausible possibilities include:

1. conditions in tropical regions may be more favourable for proliferation or transmission of microbes and pathogens^18–20^, such as the bacterium (*Brucella* spp.) that has been implicated in causing the disease^2,10^;
2. toads move around more in tropical climates than in cooler ones, thereby placing more stress on their spines even if their overall net rates of dispersal (displacement from the original point over time) is low^21^;
3. the tropical dry-season may force toads to aggregate in moist locations within the arid landscape^22–24^, promoting bacterial transmission; and/or
4. immune investment and responses may differ between individuals inhabiting tropical versus temperate climates^25^, modifying susceptibility to the pathogenic effects of *Brucella* infection.

In summary, a reanalysis of the extensive dataset collected by Bower *et al*.^3^ reinforces some of their conclusions, but changes others. The accumulating evidence of spatial variation in spinal arthritis in cane toads provides fascinating insights into the challenges posed by a biological invasion, and testifies to the advantages of collaboration between “competing” groups in clarifying the multifactorial underpinnings of biological phenomena.

## References

[1] Brown GP, Shilton CM, Phillips BL, Shine R (2007). Invasion, stress, and spinal arthritis in cane toads. Proc. Natl. Acad. Sci. USA.

[2] Shilton CM, Brown GP, Benedict S, Shine R (2008). Spinal arthropathy associated with *Ochrobactrum anthropi* in free-ranging cane toads (*Chaunus* [*Bufo*] *marinus*) in Australia. Vet. Pathol..

[3] Bower DS, Yasumiba K, Trumbo DR, Alford RA, Schwarzkopf L (2018). Spinal arthritis in cane toads across the Australian landscape. Sci. Rep..

[4] Urban M, Phillips BL, Skelly DK, Shine R (2008). A toad more traveled: the heterogeneous invasion dynamics of cane toads in Australia. Am. Nat..

[5] Brown GP, Phillips BL, Dubey S, Shine R (2015). Invader immunology: invasion history alters immune-system function in cane toads (*Rhinella marina*) in tropical Australia. Ecol. Lett..

[6] Hudson CM, Brown GP, Shine R (2016). It is lonely at the front: contrasting evolutionary trajectories in male and female invaders. R. Soc. Open Sci..

[7] Hudson CM, McCurry MR, Lundgren P, McHenry CR, Shine R (2016). Constructing an invasion machine: the rapid evolution of a dispersal-enhancing phenotype during the cane toad invasion of Australia. PLoS One.

[8] Gruber J, Brown GP, Whiting M, Shine R (2017). Is the behavioural divergence between range-core and range-edge populations of cane toads (*Rhinella marina*) due to evolutionary change or developmental plasticity?. R. Soc. Open Sci..

[9] Kosmala G, Brown GP, Christian K, Hudson CM, Shine R (2018). The thermal dependency of locomotor performance evolves rapidly within an invasive species. Ecol. Evol..

[10] Scholz HC (2016). The change of a medically important genus: Worldwide occurrence of genetically diverse novel *Brucella* species in exotic frogs. PLoS One.

[11] Rousseeuw PJ, Hubert M (2011). Robust statistics for outlier detection. WIREs Data Mining Knowl. Discov..

[12] Lindström T, Brown GP, Sisson SA, Phillips BL, Shine R (2013). Rapid shifts in dispersal behavior on an expanding range edge. Proc. Natl. Acad. Sci. USA.

[13] Allendorf, F. W. & Luikart, G. Conservation and the genetics of populations (John Wiley & Sons, 2009).

[14] Bates D, Mächler M, Bolker B, Walker S (2015). Fitting linear mixed-effects models using lme4. J. Stat. Softw..

[15] Fox, J. & Weisberg, S. An R companion to applied regression, second edition (Sage, Thousand Oaks, 2011).

[16] Alexander RM (1980). Forces in animal joints. Engineer. Med..

[17] L’Hermette MF, Tourny-Chollet C, Polle G, Dujardin FH (2006). Articular cartilage, degenerative process, and repair: current progress. Int. J. Sports Med..

[18] Altizer S, Ostfeld RS, Johnson PT, Kutz S, Harvell CD (2013). Climate change and infectious diseases: from evidence to a predictive framework. Science.

[19] Bachar A (2010). Soil microbial abundance and diversity along a low precipitation gradient. Microb. Ecol..

[20] Wu Y (2009). Changes in the soil microbial community structure with latitude in eastern China, based on phospholipid fatty acid analysis. Appl. Soil Ecol..

[21] Schwarzkopf L, Alford RA (2002). Nomadic movement in tropical toads. Oikos.

[22] Cohen MP, Alford RA (1996). Factors affecting diurnal shelter use by the cane toad, *Bufo marinus*. Herpetologica.

[23] Schwarzkopf, L. & Alford, R. A. Desiccation and shelter-site use in a tropical amphibian: comparing toads with physical models. *Funct*. *Ecol*. 193–200 (1996).

[24] Brown GP, Kelehear C, Shine R (2011). Effects of seasonal aridity on the ecology and behaviour of invasive cane toads in the Australian wet-dry tropics. Funct. Ecol..

[25] Martin LB, Pless M, Svoboda J, Wikelski M (2004). Immune activity in temperate and tropical house sparrows: a common-garden experiment. Ecology.

